# Rab GTPase Mediated Procollagen Trafficking in Ascorbic Acid Stimulated Osteoblasts

**DOI:** 10.1371/journal.pone.0046265

**Published:** 2012-09-26

**Authors:** Noushin Nabavi, Sofia Pustylnik, Rene E. Harrison

**Affiliations:** 1 Department of Cell and Systems Biology, University of Tronto Scarborought, Toronto, Ontario, Canada; 2 Department of Biological Sciences, University of Toronto Scarborough, Toronto, Ontario, Canada; Iowa State University, United States of America

## Abstract

Despite advances in investigating functional aspects of osteoblast (OB) differentiation, especially studies on how bone proteins are deposited and mineralized, there has been little research on the intracellular trafficking of bone proteins during OB differentiation. Collagen synthesis and secretion is the major function of OBs and is markedly up-regulated upon ascorbic acid (AA) stimulation, significantly more so than in fibroblast cells. Understanding the mechanism by which collagen is mobilized in specialized OB cells is important for both basic cell biology and diseases involving defects in bone protein secretion and deposition. Protein trafficking along the exocytic and endocytic pathways is aided by many molecules, with Rab GTPases being master regulators of vesicle targeting. In this study, we used microarray analysis to identify the Rab GTPases that are up-regulated during a 5-day AA differentiation of OBs, namely Rab1, Rab3d, and Rab27b. Further, we investigated the role of identified Rabs in regulating the trafficking of collagen from the site of synthesis in the ER to the Golgi and ultimately to the plasma membrane utilizing Rab dominant negative (DN) expression. We also observed that experimental halting of biosynthetic trafficking by these mutant Rabs initiated proteasome-mediated degradation of procollagen and ceased global protein translation. Acute expression of Rab1 and Rab3d DN constructs partially alleviated this negative feedback mechanism and resulted in impaired ER to Golgi trafficking of procollagen. Similar expression of Rab27b DN constructs resulted in dispersed collagen vesicles which may represent failed secretory vesicles sequestered in the cytosol. A significant and strong reduction in extracellular collagen levels was also observed implicating the functional importance of Rab1, Rab3d and Rab27b in these major collagen-producing cells.

## Introduction

Osteoblasts (OBs) are the specialized collagen producing cells of the bone tissue. Collagen is the main component of connective tissue and the most abundant protein making up between 25–35% of the body's protein content [1] and 90% of the bone tissue, with 95% of it being collagen type I. OBs undergo a sequential process during their maturation from proliferation to differentiation to mineralization. Each stage encompasses specific and well established changes in gene expression, protein expression, and cellular architecture [2], [3].

In AA-stimulated OBs, newly synthesized proteins, procollagen included, enter the biosynthetic/secretory pathway. The bulk of research on procollagen trafficking to date has been done on fibroblasts. Type I collagen is translated as α1(I) and α2(I) chains on ribosomes as pre-procollagen molecules. The chains enter the lumen of the rough endoplasmic reticulum (RER) where the C-propeptides of two α1(I) chains and one α2(I) associate to initiate triple helix folding which occurs from C-terminus to N-terminus direction [4]. The proline and lysine residues are hydroxylated [5] in the ER and these modifications are necessary for stabilization of triple helix and collagen fibril crosslinking in the extracellular space, respectively [6]. Cofactors of hydroxylation are ferrous ions, α-ketoglutarate, oxygen and AA. The 3 chains are glycosylated as that is essential for the assembly of type I collagen chains into a triple helix and final fibrillogenesis. Procollagen glycosylation is mediated by β- and α-glucosyltransferase enzymes and occurs partially in the ER [7]. Mono- and disaccharides are added through glycosidic bonds to make galactosylhydroxylysine and glucosylgalactosylhydroxylysine that contribute to the morphological variations of collagens [8]. The cofactor of glycosylation is manganese without which procollagen cannot leave the ER [4]. The rate limiting step for the folding of the triple helix is the cis-trans-isomerization of prolyl peptide bonds in the α-chains, which is catalyzed by the enzyme peptidyl-prolyl cis-trans-isomerase (PPI) [9]. The procollagen trimer is further assembled and stabilized by many enzymes such as protein disulphide isomerase (PDI) and HSP47 in the ER [10], [11]. PDI, in addition to its role in disulfide formation, serves as the proline hydroxylase, as well as recognizing consensus sequences for N-linked glycosylation on nascent collagen chains [12], [13], [14]. Other RER enzymes such as hydroxylases, glycosyltransferases, isomerases, and several other chaperones such as Binding immunoglobulin Protein (BiP) assist in the post-translational modification, folding and processing of the procollagen molecules [1].

Procollagen containing vesicles (60–80 nm in diameter) are transported to the Golgi complex with the aid of trafficking molecules such as COPI, COPII, and dynamin [15], [16], [17] traversing the Golgi stacks in small transport vesicles. Kadler and colleagues showed through dual fluorescence and scanning electron microscopy (SEM) that procollagen is directed to the PM in pleiomorphic Golgi-to-PM carriers without ever leaving the Golgi complex [18], supporting the cisternal maturation model [19], [20]. The pleiomorphic collagen vesicles were measured by Kadler to be 0.5–1.7 µm long and 28-nm in diameter. O-linked glycosylation of procollagen occurs in the Golgi [21] in which they are further packaged in secretory vesicles for secretion [22]. Cargo sorting reaches a high level of complexity and sophistication at the trans-Golgi network.

Large and processed procollagen carriers (300–500 nm in diameter) have been shown to fuse with the PM in fibroblasts and are subsequently extruded out of cells and form a fibripositor (fibril depositors) in which collagen fibers fuse and elongate to form fibrils [16], [18], [19], [20]. Fibripositors have never been recorded in OB and therefore their presence in OBs is still controversial. Electron microscopy images of procollagen containing vesicles being released to the ECM in fibroblasts show electron dense secretory vesicles that are continuous with the Golgi complex and appear to be associated with microtubules (MTs) [23]. This transport is further shown to be MT dependent as nocodazole treatment results in improper fibrillogenesis outside the cells.

We were particularly interested in deciphering the molecular machinery driving procollagen trafficking in stimulated OBs. In this study, we explored the role of Rab proteins in procollagen vesicle trafficking in AA-stimulated OBs. Rab proteins are GTPases that function as a molecular switch by cycling between a GDP-bound inactive state and a GTP-bound active state [9]. Different Rab GTPases are localized to the cytosolic face of specific intracellular membranes, where they function as regulators of distinct steps in membrane traffic pathways [9]. For instance, previous studies in other cell types have shown that Rab1 co-localizes with vesicles being transported from the endoplasmic reticulum (ER) to the Golgi, Rab 8 is implicated in vesicle trafficking from Golgi to plasma membrane (PM), Rab11 moves cargo from recycling endosomes to PM, and Rab5 and 7 are involved in endocytic trafficking [10]. However, the mechanism behind procollagen cargo trafficking and sorting in osteogenic cells is not known and the aim of this study was to examine which Rab GTPases mediate procollagen trafficking in OBs.

## Results

### Microarray determination of up-regulated Rab GTPases during OB differentiation

**Figure 1:**
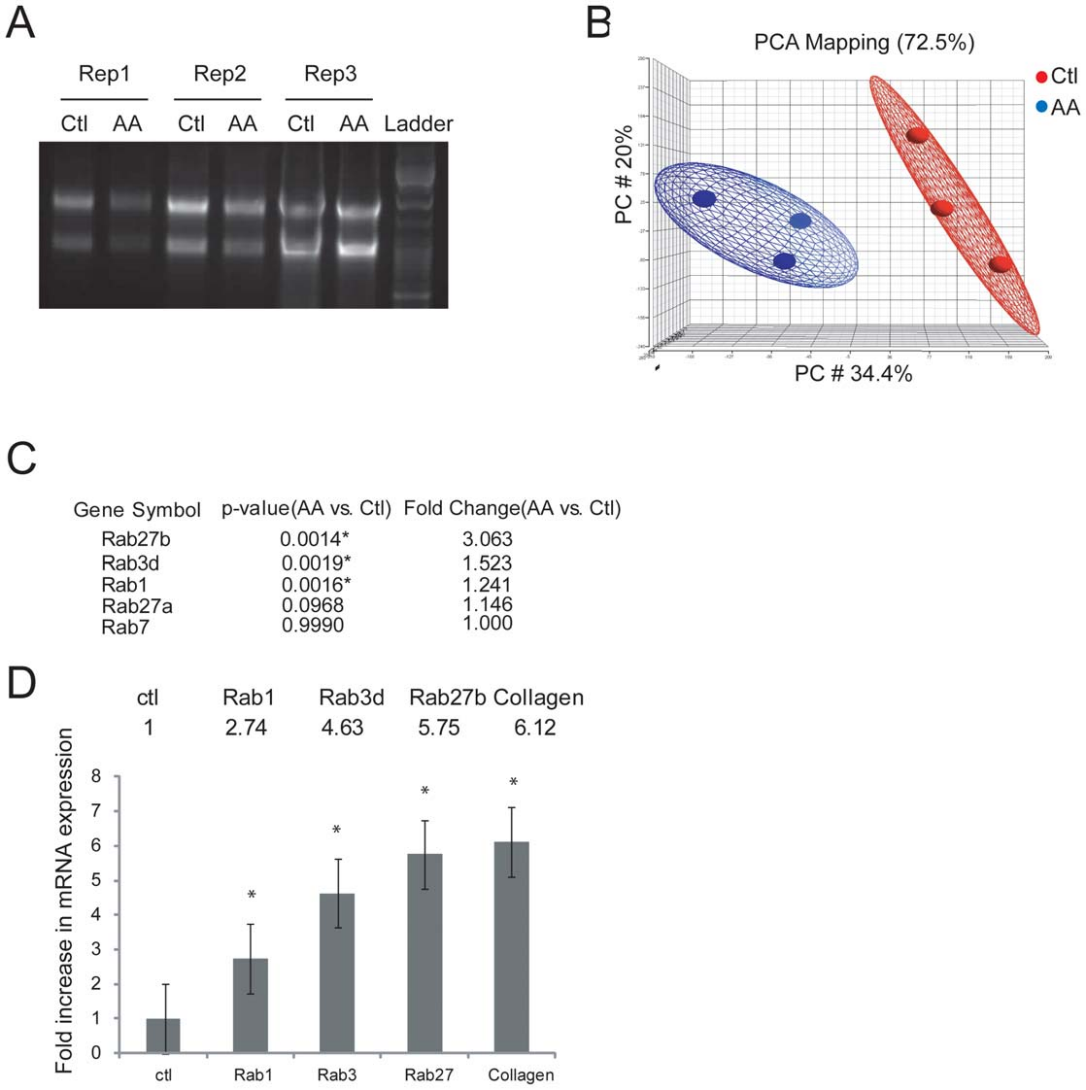
mRNA expression analysis of control and 5-day AA-treated MC3T3-E1 OBs using microarray analysis. (A) Quality of extracted RNA from 3 replicates of control and AA-treated cells assessed on a 1% agarose gel. (B) Principal Component Analysis (PCA) plot of control and AA-treated OB genes showed that the two treatments are in two distinct regions and therefore independent of one another. This plot further confirmed the independent gene expression in the two treatments. (C) List of up-regulated Rab GTPase transcripts in differentiating OBs compared to control OBs. * indicates genes that were significantly up-regulated. (D) qRT-PCR validation of mRNA expression levels of the three Rab GTPases that were significantly up-regulated in 5-day AA treated cells compared to undifferentiated control cells. The Y-axis represents the fold change in expression compared to controls in triplicate experiments. * p<0.05.

Initially, we were interested in determining the molecular machinery driving procollagen vesicle trafficking in OBs. To identify the endogenous Rab GTPases and their expression levels in differentiating OBs, Affymetrix microarray technology was utilized. MC3T3-E1 cells were stimulated with AA, a classic co-factor required for hydroxylation of proline and lysine residues in collagen [24]. AA also induces OB differentiation as evidenced by dramatic increases in the expression of many bone related genes and proteins, notably collagen Type I [25]. Total RNA from triplicate experiments was isolated and checked for purity and degradation on 1% agarose gel (shown in Figure 1A) as well as for 260∶280 O.D. ratio. The RNA was then sent to be hybridized onto Affymetrix mouse 430 2.0 microarray chips under high stringency conditions and scanned. Arrays were inspected for obvious contamination or noticeable differences, defects, and overall brightness. The intensity of each spot on the array chip was translated by microarray software (Partek Inc.) into numbers, which provided a large amount of information about the array, including the mean, median, and standard deviation of the foreground and background intensities of each spot as well as the perfect match and mismatch probe intensities. The complete array is available in the GEO database (accession number: GSE37676). The Principle Component Analysis (PCA) plot in Figure 1B visually summarizes the correlation between the control and AA-treated cells in a reduced multi-dimensional matrix, generated by performing a covariance analysis between the two populations. Data from control triplicates presented in three circles on the left side of the plot are spatially separated with no overlap with the differentiated triplicates on the right. Level of differentiation is separated along x-axis increasing from left to right. Of a total of 45,103 genes identified on the PCA scatter plot, 22,696 were up-regulated and 22,407 genes were down-regulated. A balanced differential expression of 1.5 fold or higher was taken to be significant. Several Rab GTPases were up-regulated by AA-stimulation, using the differential expression parameter on Partek software (Figure 1C). We were particularly interested in Rab1, Rab3d, and Rab27b as they were exclusively up-regulated in our analysis and have been previously implicated in playing roles in biosynthetic/secretory transport in other cell types [26], [27], [28], [29], [30]. Additionally, our microarray analysis did not identify any other Rabs (including Rab7 or Rab27a; Figure 1C) or Rab guanine exchange factors (GEFs) or GTPase activating proteins that were significantly up-regulated upon AA-stimulation (not shown). The up-regulation of Rab1, 3d and 27b was first validated using qRT-PCR: Rab1, Rab3d and Rab27b mRNA expression was significantly increased 2.7-fold, 4.6-fold, and 5.7-fold, respectively in 5 day AA-differentiated OBs compared to undifferentiated control cells as shown in Figure 1D. Thus OB differentiation includes enhanced expression of trafficking machinery in addition to a surge in secretory cargo.

### Analysis of intracellular trafficking of procollagen in osteoblasts following AA stimulation

**Figure 2:**
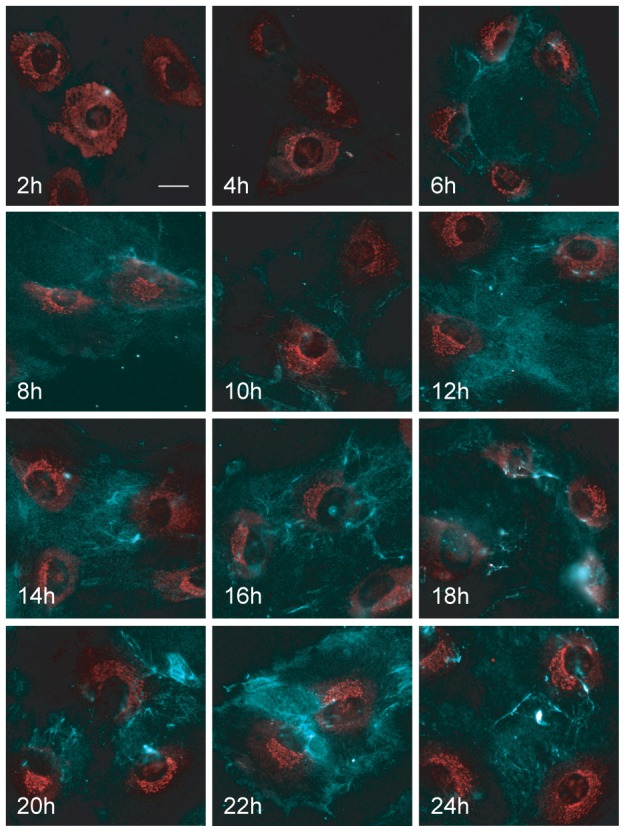
Time-course of movement of procollagen in AA-differentiating MC3T3-E1 cells. Cells were fixed and immunostained for extracellular procollagen (in blue). The same cells were then permeabilized using Triton X-100 and were immunostained for internal procollagen as well as secreted collagen (both shown in red) since the antibody recognizes both procollagen and mature forms. The panels depict cells fixed at 2, 4, 6, 8, 10, 12, 14, 16, 18, 20, 22, and 24 hours after AA addition. Intracellular procollagen was visible in the Golgi as early as 4 hours after AA stimulation and extracellular procollagen was visible at 6 hours. The 6 hour AA treatment was selected for further experiments as sufficient for procollagen translocation from ER to Golgi to the PM for secretion was observed at this timepoint. Scale bars, 10 µm.

We next wanted to determine the optimal time points to study trafficking of procollagen within the biosynthetic pathway following OB stimulation. As procollagen expression is markedly up-regulated in OBs upon addition of AA, we examined the minimum time for procollagen appearance and movement from its site of synthesis in the ER to the trans-Golgi network and PM for secretion. MC3T3-E1 cells were passaged onto coverslips and treated with collagenase to remove any contaminating basal extracellular collagen. Cells were either untreated or treated with AA for 2, 4, 6, 8, 10, 12, 14, 16, 18, 20, 22, or 24 hours. Cells were then stained for extracellular collagen followed by permeabilization and immunostaining for total collagen with the same antibody. The collagen antibody recognizes both intracellular procollagen as well as mature secreted collagen and depending on whether the cells were permeabilized or not, we could detect both the intracellular procollagen as well as the secreted collagen or exclusively detect extracellular mature forms. Epifluorescence imaging revealed that procollagen had translocated to a Golgi-like structure starting at 4 hours, and by 8 hours of AA-treatment it was strongly visible on the cell surface (Figure 2). Further incubation times revealed a marked accumulation of an extracellular collagen network encompassing the cell monolayer (Figure 2).

**Figure 3:**
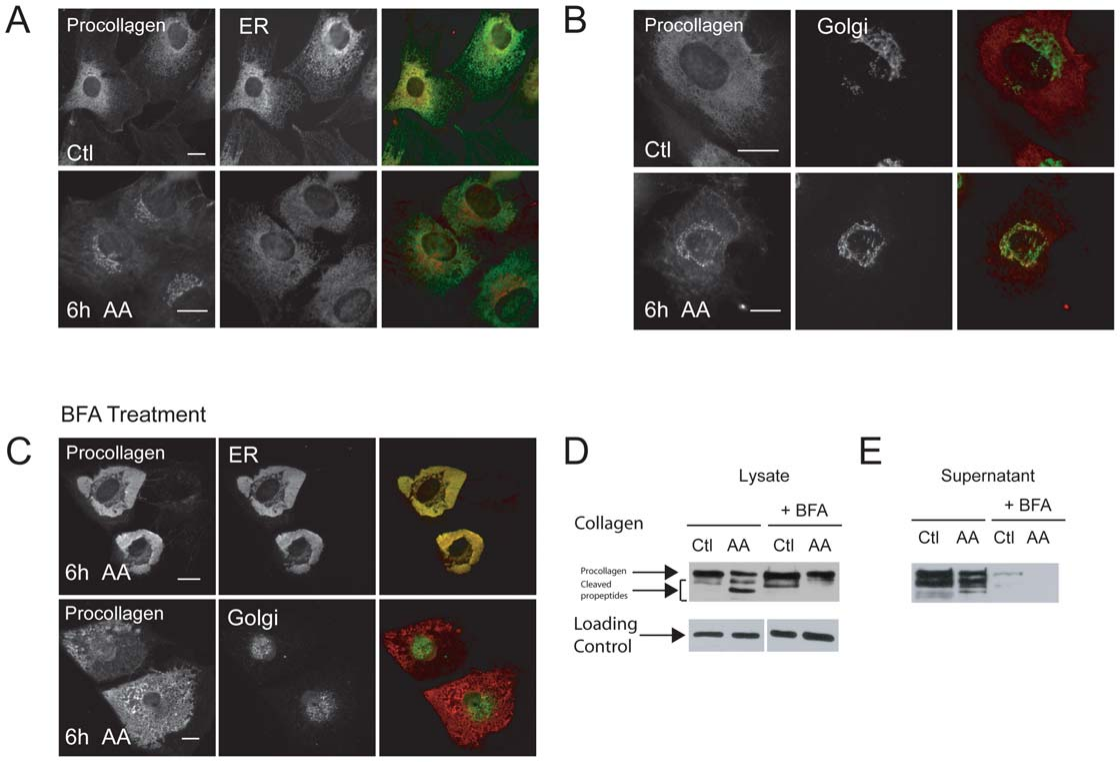
Procollagen localizes to the ER and translocates to the Golgi after AA-stimulation of OBs. (A) Control and 6 hour AA differentiated MC3T3-E1 cells were fixed and stained with procollagen antibody shown in red and ER (PDI) antibody in green. (B) Same experiment as (A) except that the Golgi (GM130) was immunostained in green. Procollagen resides in the ER in undifferentiated control cells and upon addition of AA is translocated to the Golgi. (C) Treatment of cells with 0.5 µg/ml of Brefeldin A overnight disassembles the Golgi and causes the accumulation of procollagen (red) in the ER (green) and inhibits its transport to Golgi in AA-stimulated OBs. Scale bars, 10 µm. (D–E) Collagen immunoblots of control and AA-treated cell lysates and their conditioned media. The higher band on the blot represents the partially mature basal procollagen residing in the ER (as most of the procollagen modification occurs in ER) while the lower bands likely represent the cleaved procollagen peptides. The lower band appeared after AA addition presumably after processing of procollagen in the Golgi. Treatment of cells with BFA disrupted procollagen modifications in Golgi and therefore only the higher procollagen band was apparent in the immunoblot, presumably the procollagen in the ER. (E) BFA treatment also inhibited both basal and processed collagen secretion in the conditioned media collected from control and AA-stimulated cells as shown in the immunoblot.

We then determined more precisely the movement of procollagen through the biosynthetic pathway following AA-stimulation by immunostaining cells for procollagen and either ER or Golgi markers at various time points post stimulation. The ER, by itself, appeared large and dispersed throughout the cytoplasm in both control and AA-differentiated cells (Figure 3A). In control (unstimulated) MC3T3-E1 cells, procollagen was found within the ER at a higher fluorescence intensity compared to 6 hour AA differentiated cells (Figure 3A). In cells stimulated with 6 hours with AA, procollagen was instead enriched in the Golgi and strongly co-localized with Golgi markers by immunofluorescence (Figure 3B). The Golgi complex morphology in both control and differentiated cells comprised of multiple stacks, vesicles, and small fibrillar structures (Figure 3B). Procollagen ER-Golgi transport in AA-stimulated cells was confirmed by BFA treatment to disassemble the Golgi [31], resulting in a Golgi disruption and inhibition of procollagen transport from ER to Golgi [31] in AA-treated cells, shown in Figure 3C. The diminished and fragmented Golgi phenotype we observed and the collapse of Golgi cargo back to the ER is consistent with other studies in the literature [32], [33], [34].

The movement of procollagen through the biosynthetic pathway following stimulation with AA was confirmed biochemically. Western blotting of lysates from control and AA-treated cells showed a prominent band at 190 kDa in control cells and an appearance of 2 lower bands in AA-treated cells (Figure 3D). Following procollagen synthesis and assembly in the ER, the oligosaccharide chains of the trimer are modified in the Golgi [35] where the N and C-propeptides are cleaved either before secretion upon interacting with the PM or shortly after secretion [36], [37]. The lower bands in the AA-treated cell lysates likely indicate the mature cleaved products that have moved through the Golgi upon AA stimulation. To support this, treatment of cells with BFA caused a disappearance of the lower MW bands in AA treated cells (Figure 3D), suggesting perhaps the accumulation of procollagen in the ER and inhibition of collagen processing in the Golgi. BFA also inhibited collagen secretion as previously described [31] as shown by immunoblotting of control and AA-treated supernatants (conditioned media) collected from cells treated with BFA, compared to untreated cells (Figure 3E).

### Colocalization of Rab1, 3d and 27b with procollagen compartments in MC3T3-E1 OBs

**Figure 4:**
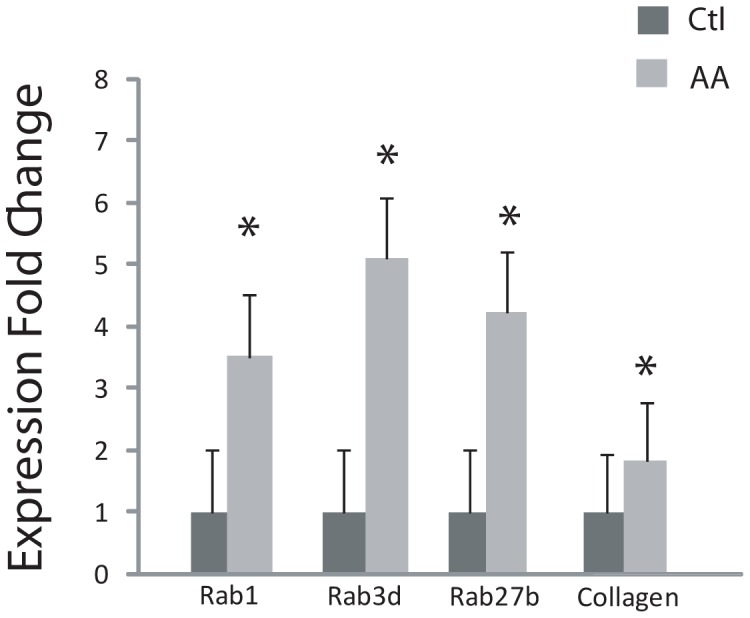
qRT-PCR analysis of mRNA expression levels of three identified Rab GTPases in OBs treated with AA for 6 hours. Quantitative PCR analysis was performed on mRNA from control and 6 hour AA-stimulated MC3T3-E1 osteoblasts. The graph shows the signal intensity of OB Rab GTPase expression normalized against that of GAPDH from 3 independent experiments. Data is reported as mean ± SEM A significant increase in Rab1, 3d, and 27b mRNA expression levels after 6 hours of AA treatment versus no stimulation of control cells. * p<0.05.

**Figure 5:**
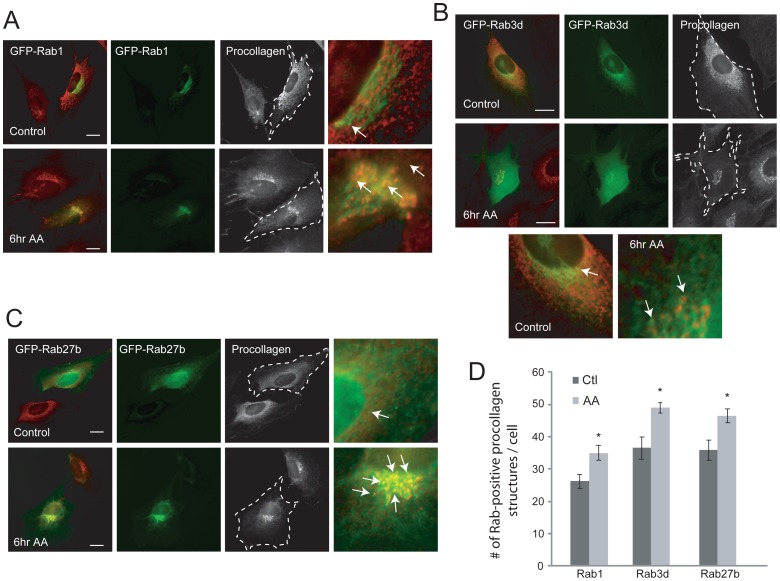
Rab GTPases partially colocalize with procollagen-containing compartments in differentiating OBs. (A) Immunofluorescent images of MC3T3-E1 cells transfected with Rab1-GFP overnight and differentiated for 6 hours with AA. Cells were fixed and immunostained with procollagen shown in red. (B–C) Cells were transfected with Rab3d and Rab27b-GFP and immunostained with a procollagen antibody. Arrows point to the Rab-positive procollagen containing vesicles and the dotted lines point to the transfected cells in black and white procollagen images. The last panels show a magnified image of a select region showing partial co-localization of the Rabs with procollagen. Scale bars, 10 µm. (D) Quantification of the number of co-localized Rab GTPase and procollagen containing vesicles using weighted colocalization LSM software in 30 confocal images. The graph depicted a significant increase in all three Rab GTPases association with in AA-differentiated OBs compared to undifferentiated control cells. The Y-axis represents the number of Rab-positive procollagen containing vesicles and * p<0.05. Data is expressed as mean ± SEM from 3 independent experiments (n = 20).

Once we had established the minimum time for procollagen mobilization after AA-stimulation, we proceeded to analyze the role of Rab GTPases in this process. We detected extracellular procollagen as early as 6 hours after AA-exposure so we first determined whether Rab expression was also acutely up-regulated. Quantitative RT-PCR showed that even at 6 hours of AA-stimulation the expression of Rab1, 3d, and 27b were significantly increased compared to levels in unstimulated cells (Figure 4). To visualize Rab distribution in these cells, MC3T3-E1 OBs were transiently transfected with GFP chimeras of wild-type Rab1, Rab3d and Rab27b. After overnight transfection, cells were treated with AA for 6 hours and then fixed and stained for collagen and imaged with confocal microscopy (Figure 5). Cells were also dually immunostained with antibodies to ER and Golgi resident proteins to observe Rab localization in these organelles (not shown). Rab1-GFP showed partial co-localization with procollagen in resting OBs, which was increased in AA-stimulated cells (Figure 5A, D). Rab1-GFP showed almost exclusive overlap with the Golgi apparatus (not shown), which has been observed in other cell types [26], [38]. Similar to Rab1, Rab3d-GFP and Rab27b-GFP also showed considerable colocalization with the Golgi marker, GM130, and showed a similar enhanced colocalization with procollagen in AA-stimulated cells, compared to control cells (not shown). Additionally, Rab3d-GFP and Rab27b-GFP localized more abundantly to the cytosol, including along dispersed vesicles at the cell periphery (Figure 5B–D) regardless of expression level, which was not observed with Rab1-GFP (Figure 5A–C). In other studies, Rab3 isoforms have been shown to localize to exocytic vesicles in endocrine and neuronal cells [39] as well as playing a role in early vesicle biogenesis and Golgi trafficking in rat epithelial tissues [29]. Similar to Rab3, Rab27 has been shown to play a role in epithelial cells as well as synaptic vesicle transport in neurons [40]. The differential localization of Rabs suggests their specificity in transport of vesicles in the secretory pathway. In this study, Rab3d-GFP and Rab27b-GFP localized to small discrete vesicles more often than Rab1-GFP that may represent secretory procollagen containing vesicles and this localization was observed to increase in AA-differentiated OBs (Figure 5A–D). The number of discrete Rab1, Rab3d, and Rab27 vesicles that colocalized with procollagen per cell was quantified (Figure 5D). It is noteworthy that 6 hour AA-differentiation of cells markedly increased Rab3d-GFP and Rab27b-GFP association with distinct procollagen structures particularly at the peripheral regions of cells. The increased colocalization of Rabs observed with procollagen could be due to the requirement of these stimulated cells to accommodate the increasing levels of procollagen and assist in their trafficking.

### Role of identified Rab GTPases on collagen trafficking in OBs

**Figure 6:**
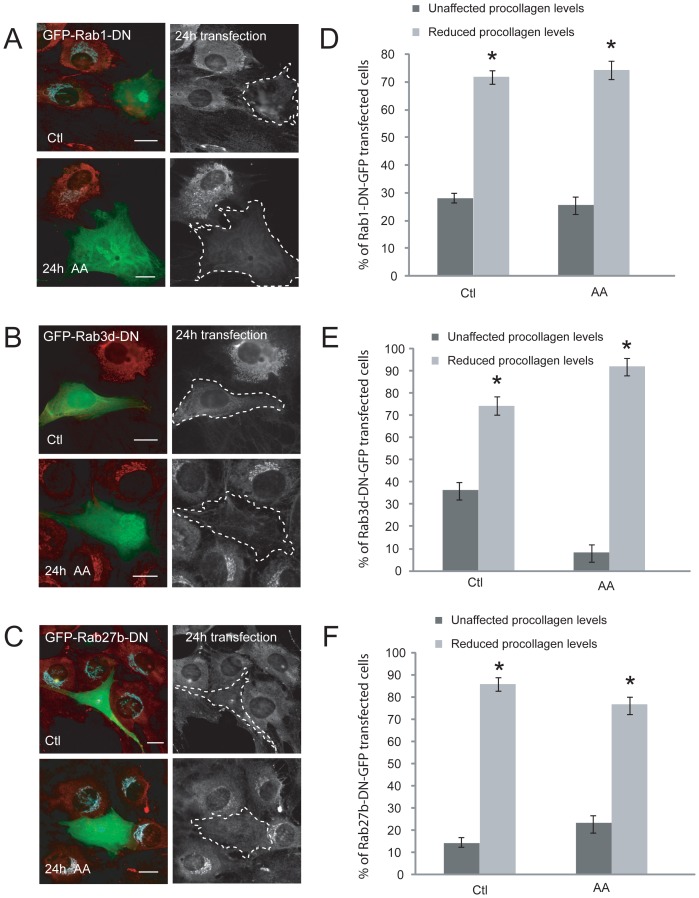
Effect of mutant Rab GTPase expression on procollagen protein levels in control and AA-stimulated OBs. (A) MC3T3-E1 cells were transfected with Rab1-DN-GFP overnight and differentiated for 6 hours with AA, then fixed and immunostained for procollagen shown in black and white (red in merged images) and Golgi in blue. The dotted lines outline the transfected cells. (B–C) Cells were transfected with DN Rab3d and Rab27b-GFP overnight respectively, and immunostained with a collagen antibody. Scale bars, 10 µm. (D–F) Graphs depict the percentage of mutant Rab cells in both control and AA-treated cells that showed a significant decrease in total procollagen immunostaining, relative to untransfected cells within the same field of view, * p<0.05. Data is expressed as mean ± SEM from 3 independent experiments (n = 100).

To decipher the roles of the up-regulated Rab1, Rab3d, and Rab27b on procollagen trafficking, Rab DN constructs were utilized. These Rabs were mutated by replacing tyrosine 22 with aspargine (T22N) or serine22 with aspargine (S22N) to cause reduced affinity for GTP binding [41]. MC3T3-E1 cells were passaged onto glass coverslips using trypsin and treated with collagenase to remove basal secreted collagen, and then transfected overnight with either of the DN Rab constructs (Rab1, Rab3d, and Rab27b-GFP). Control cells and cells treated with AA for 6 hours were then fixed and immunostained with a collagen antibody. Epifluorescence imaging of these cells revealed a marked reduction in total procollagen levels in all Rab-DN-GFP transfected cells, compared to untransfected control cells (Figure 6A–C). This occurred in transfected, control cells with Rab3d-GFP expressing cells showing an additional pronounced procollagen phenotype following AA-stimulation. We further attempted to confirm these results with siRNA knockdown of these Rabs; however the absence of suitable antibodies for this cell type precluded confirmation of siRNA knockdown efficiency and identification of siRNA-transfected cells. Total procollagen protein levels in RabDN-GFP transfected cells were quantified and scored for normal (unaffected) procollagen production or reduced (disrupted) procollagen levels, compared to untransfected cells. The graph in Figure 6D depicts the percentage of transfected control and AA-treated cells that showed normal versus reduced collagen levels. It was observed that approximately 70% of both control and AA-treated cells transfected with Rab1-DN-GFP showed significantly reduced total procollagen immunostaining, compared to untransfected cells. An even more striking trend was observed in cells transfected with Rab27b-DN-GFP, where 80–90% of cells had nearly absent procollagen protein levels upon mutant Rab expression (Figure 6F). Over 90% of AA-treated Rab3d-DN-GFP transfected cells also had dramatically reduced total collagen levels, while less than half of unstimulated transfected cells only showed a similar defect (Figure 6E). These results revealed an important role of Rab1, 3d and 27b in procollagen homeostasis in osteoblasts.

### Mechanism of reduced total procollagen production in MC3T3-E1 OBs expressing mutant Rabs

**Figure 7:**
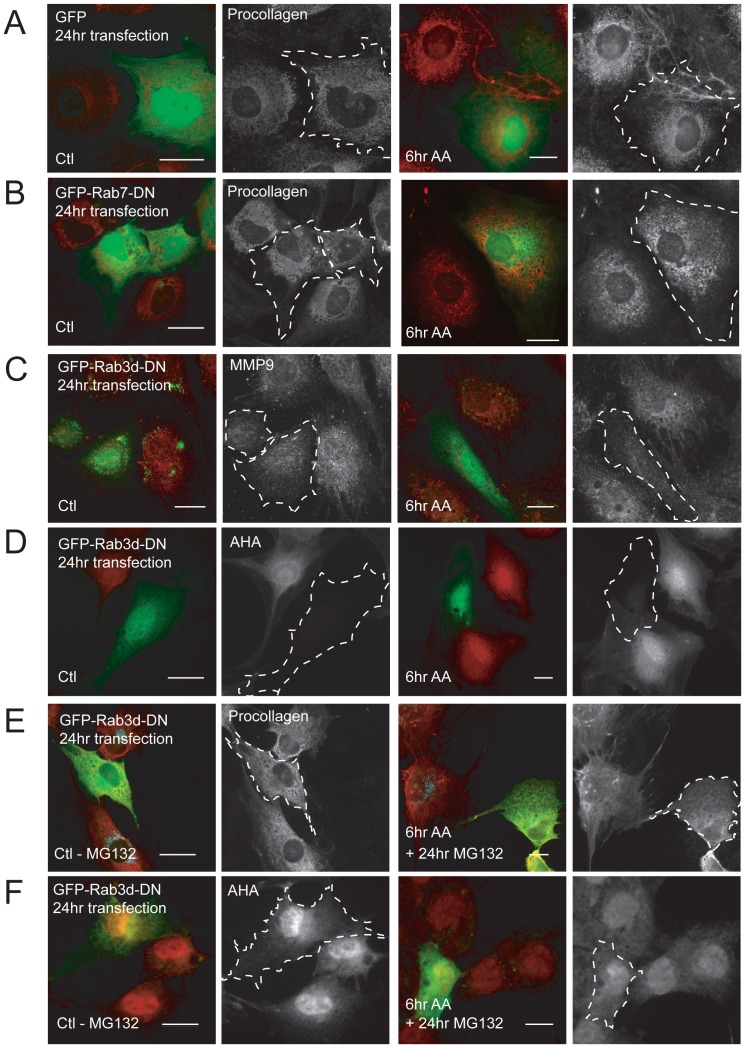
Investigating the mechanism behind reduced procollagen production in Rab3d-DN-GFP expressing OBs. (A–B) GFP alone and Rab7-DN-GFP transfections did not change total procollagen levels (shown in black and white, or red in merged images) in control and AA-stimulated OBs. Dotted lines in black and white images point to transfected cells. (C) Control and 6-hour AA treated cells were transfected with Rab3d-DN -GFP and fixed and immunostained with MMP9 antibody shown in black and white (red in merged images). MMP9 staining was significantly lower in the transfected cells compared to untransfected control cells in both control and AA-treated cells. (D) Protein synthesis was shut down in both control and AA-treated cells transfected with Rab3d-DN-GFP overnight. This was measured using an AHA protein synthesis labelling kit. (E) Cells were transfected with Rab3d-DN-GFP and treated with MG132, a proteasome inhibitor, overnight. During this time, one population of cells was stimulated with AA for 6 hours. Cells were then fixed and immunostained with procollagen antibodies and imaged. Immunofluorescent images reveal that MG132 treatment restores procollagen intensity levels in Rab3d-DN cells, in both control and AA-treated cells. (F) Protein synthesis was rescued in both control and AA-treated cells transfected with Rab3d-DN-GFP overnight when cells were treated with MG132, as detected by AHA labelling. Scale bars, 10 µm.

It was clear that down-regulation of the three investigated Rab proteins had profound changes on cellular procollagen levels. We sought to identify the mechanism behind disrupted procollagen levels in these cells. MC3T3-E1 OBs were transfected overnight with an empty GFP construct to determine if expression of the fluorescent protein impacted collagen production/stability. OBs were then stimulated or not with AA for 6 hours and fixed and immunostained for collagen. Procollagen intensity did not change in GFP-alone transfected cells compared to untransfected cells (Figure 7A). To investigate whether this was a general effect of DN Rab expression, MC3T3-E1 cells were transfected overnight with Rab7-DN-GFP, a Rab specific to the endocytic pathway [42] that was not up-regulated upon AA-stimulation (Figure 1C, Figure S1A), prior to collagen immunostaining. Both control and AA-treated OBs expressing Rab7-DN-GFP showed similar procollagen levels to that of untransfected cells (Figure 7B).

We next investigated whether the mutant Rab expression caused a general disruption in biosynthetic protein production/trafficking. Rab3d-DN-GFP was chosen for the rest of these studies as it had the most pronounced effect on procollagen protein levels in AA-treated OBs. We looked at the effects of overnight expression of Rab3d-DN-GFP on the production of matrix metalloprotease-9 (MMP-9), a known protein in the biosynthetic pathway that is constitutively expressed in OBs [43]. Total MMP-9 levels were reduced, particularly in AA-treated OBs expressing Rab3d-DN-GFP, compared to untransfected control cells (Figure 7C). Thus it appeared that interference of trafficking machinery selective to the biosynthetic pathway impacted general cellular protein production. Due to the low transfection efficiency of these constructs we were unable to assess whether secretory proteins were down-regulated at the mRNA level. Instead we looked at total protein production in mutant Rab expressing cells, using AHA, a fluorescent methionine analog that becomes incorporated into newly synthesized proteins [44]. Total de novo protein synthesis was completely shut down 24 hours post transfection of mutant Rab3d-GFP (Figure 7D). Thus strong negative feedback loops seemed to be at play when movement through the biosynthetic pathway was manipulated, resulting in an arrest of global protein production. Still, the disappearance of the pre-existing procollagen (prior to transfection) in these RabDN-transfected cells could not be explained. Either procollagen was cleared by secretion or was being selectively degraded upon RabDN-GFP expression. To determine if proteasomes were involved in pre-existing procollagen removal, Rab mutant expressing cells were treated with 1 µM of the proteasome inhibitor, MG132, for 6 and 24 hours. Proteasome inhibition rescued protein synthesis and procollagen levels in cells expressing Rab-DN overnight. Figure 7E shows the visible intracellular procollagen in cells expressing mutant Rab3d overnight in the presence of MG132. Additionally, in the absence of proteasome activity, protein synthesis was also restored in Rab3d-DN-GFP transfected cells, as revealed by AHA labelling, to near normal levels comparable to untransfected cells (Figure 7F). Thus proteasome activity in addition to attenuated protein synthesis is involved in reducing collagen levels in cells expressing mutant Rab constructs overnight.

### Acute mutant Rab1, 3d, 27b expression alters procollagen trafficking through the biosynthetic pathway in OBs

**Figure 8:**
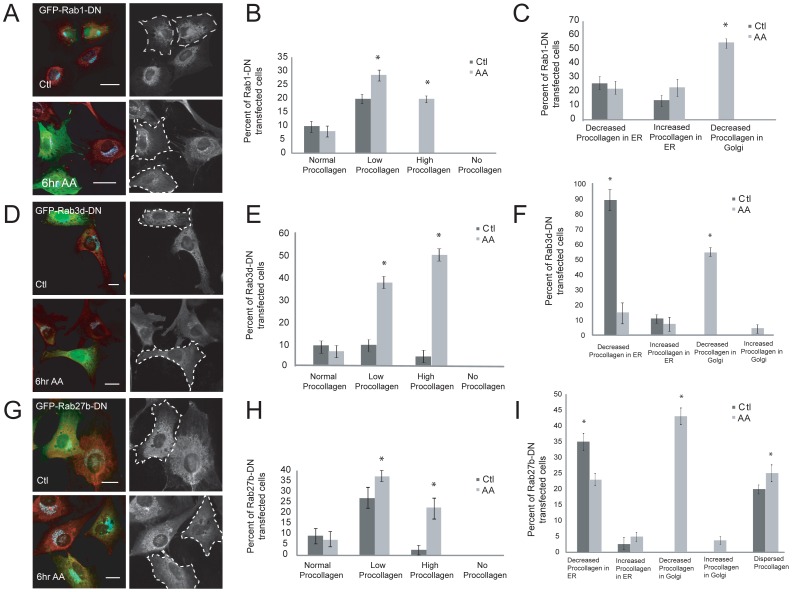
Localization of procollagen in AA-stimulated OBs transfected with mutant Rab GTPases for shorter time periods. (A) To determine the role of Rab mutants on intracellular procollagen localization and expression, control and 6 hour AA- treated cells were transfected for 6 hours with Rab1-DN-GFP, fixed, and stained for procollagen shown in red. Cells were also immunostained with PDI or GM130 to label ER or Golgi, respectively. (B) Categorization and quantification of procollagen levels in Rab1-DN transfected cells compared to untransfected control cells. (C) Localization of collagen in the ER and Golgi organelles in control and AA-treated cells that were transfected with Rab1-DN for 6 hours. Percentage of control cells showing reduced procollagen levels in ER is increased. Also, there is a significant reduction in collagen localization in Golgi in AA-treated transfected cells. (D) Control and 6 hour AA-treated cells were transfected for 6 hours with Rab3d-DN-GFP, fixed, and stained for intracellular procollagen shown in red (E) Procollagen level categorization and quantification in transfected cells compared to untransfected. (F) Quantification of percentage of cells showing differential procollagen levels in ER and Golgi in transfected cells. (G) Control and 6 hour AA- treated cells transfected for 6 hours with Rab27b-DN-GFP show a significant reduction in procollagen level intensities. (H) Categorization and quantification of differential procollagen levels in Rab27b-DN transfected cells compared to untransfected controls. (I) The graph represents the collagen intensities in ER and Golgi in Rab27-DN expressing cells quantified using ImageJ. There are a significant number of control cells presenting with reduced procollagen levels in ER. AA-treated cells also showed reduced procollagen levels in Golgi. There is also a significant increase in dispersed procollagen containing vesicles that did not label with either ER or Golgi markers in both control and AA-treated transfected cells. Scale bars, 10 µm. All data are reported as mean ± SEM from 3 independent experiments (n = 100).

To avoid activating quality control pathways and to image potential trafficking defects caused by mutant Rab expression, we drastically reduced the transfection times of these constructs. It was determined that a 6 hour transfection, combined with GFP immunostaining to amplify the GFP signal, was the minimum time necessary to visualize expression of these constructs over background fluorescence. For all experiments, cells were passaged with trypsin and treated with collagenase prior to plating. Cells were then immediately transfected with the Rab-DN constructs and stimulated, or not, with AA for 6 hours. Cells were then fixed and immunostained for GFP and collagen. Procollagen protein levels were no longer completely abolished in control or AA-treated cells transfected with Rab1-DN-GFP, compared to untransfected cells (Figure 8A). The overall cellular levels of procollagen in Rab1-DN-GFP transfected cells were quantified and compared to neighboring untransfected cells within the same field. There were significantly more Rab1-DN-GFP transfected cells that showed lower overall levels of collagen in the differentiated cells, compared to untransfected cells (Figure 8B). This may be a result of the initiation of quality control mechanisms to stop the biosynthetic pathway in differentiating cells, although no mutant Rab1 transfected cells showed “no or negligible procollagen staining” (Figure 6D) which was characteristic in cells expressing this construct overnight. A greater percentage of AA-treated Rab1-DN-GFP expressing cells showed increased procollagen staining, compared to untransfected neighbouring cells (Figure 8B). To determine if this was a result of procollagen protein being blocked from moving through the ER-Golgi pathway, cells were stained with either ER or Golgi markers. About 20% of control and AA-stimulated Rab1-DN-GFP transfected cells had reduced procollagen in the ER, compared to untransfected cells (Figure 8C). These low-procollagen expressers could again be due to activation of proteasome/inhibition of translation feedback mechanisms. Approximately 20% of AA-treated Rab1-DN-GFP transfected cells that showed an increase in procollagen within the ER, compared to untransfected control cells (Figure 8C). Potentially related to this, over 50% of Rab1-DN-GFP cells stimulated with AA showed decreased procollagen levels in the Golgi (Figure 8C), which suggest that Rab1 is important for ER to Golgi trafficking of procollagen in OBs that have been stimulated by AA.

A similar analysis was performed in cells acutely expressing Rab3d-DN-GFP and Rab27b-DN-GFP (Figures 8D–I). Cells were transfected for 6 hours and immunostained for collagen (Figures 8D and 8G). Rab3d-DN-GFP transfection resulted in a subset of cells with lower total collagen than untransfected cells (Figure 8E). A similar result was observed in cells following Rab27b-DN-GFP transfection (Figure 8H) which may be due to altered translational output in these cells, described in Figure 6. We observed a visible increase in procollagen in some cells expressing either Rab3d-DN-GFP or Rab27b-DN-GFP, compared to untransfected cells in the same field of view (Figure 8E, 8H). To see if this was a result of congestion in trafficking along the secretory pathway we additionally immunostained cells for Golgi and ER markers to determine the fate of procollagen in these mutant Rab expressing cells (Figure 8D, 8G). A very large fraction of unstimulated Rab3d-DN-GFP transfected cells had reduced procollagen in ER (Figure 8F) suggesting an early initiation of quality control mechanisms within biosynthetic pathway. Interestingly, 60% of AA-treated mutants were also quantified to have decreased collagen levels in Golgi suggesting an impairment in trafficking from ER to Golgi or an inhibition in collagen production altogether (Figure 8F). A small fraction of Rab3d-DN-GFP transfected cells had increased procollagen within the Golgi compared to untransfected cells, suggestive of a post-Golgi trafficking impairment in otherwise secretory OBs. Inspection of procollagen distribution in Rab27b-DN-GFP transfected cells also showed decreased ER-localization of procollagen in both control and AA-stimulated cells suggestive of reduced procollagen synthesis (Figure 8I). AA-stimulated cells expressing Rab27b-DN-GFP also showed procollagen levels reduced in Golgi in many cells, and a small fraction of cells with unusually high levels of Golgi-localized procollagen, compared to untransfected neighboring cells (Figure 8I) suggestive of further trafficking defects. Interestingly, expression of Rab27b-DN-GFP induced a high fraction of cells to contain ‘dispersed’ collagen vesicles of unknown origin (Figure 8I), which may represent post-Golgi vesicles that were not successfully secreted.

### Reduction in extracellular collagen levels in AA-stimulated OBs expressing mutant Rab1, 3d and 27b

**Figure 9:**
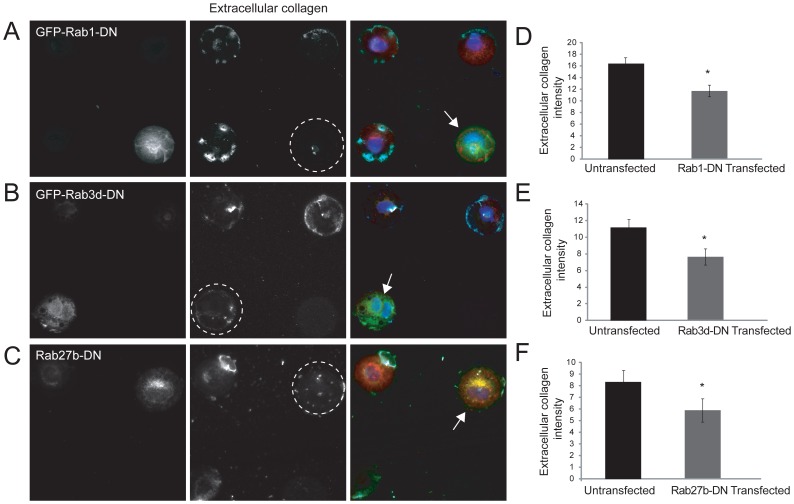
Reduced extracellular collagen secretion levels in AA-stimulated MC3T3-E1 OBs expressing mutant Rab GTPases. Cells were plated on CYTOO coverslips for 1 hour, transfected with Rab1-DN-GFP (A), Rab3d-GFP (B) or Rab27b-GFP (C) for 9 hours during which they were differentiated with AA for 8 hours. Cells were then fixed and immunostained with extracellular collagen in cyan and total procollagen shown in red. Nuclei were stained with DAPI (blue). The dotted circles in black and white images point to extracellular collagen in transfected cells. Scale bars, 10 µm. (D–F) Graphs present the mean collagen intensity levels in cells expressing mutant Rabs for 9 hours compared to untransfected cells, which was quantified using ImageJ. Expression of all three mutant Rabs resulted in a significant reduction in extracellular collagen secretion. * p<0.05. All data are reported as mean ± SEM from 3 independent experiments (n = 50).

To see if trafficking defects induced by expression of mutant Rab1, 3d and 27b translated to reduced secretion of collagen, we looked at extracellular collagen in mutant Rab expressing cells. Traditional plating of cells resulted in a web-like network of collagen (see Figure 3 for example) which made it difficult to decipher which cells were the source of the ECM. To troubleshoot this, CYTOO poly-L-lysine micropattern grids [45] were utilized to spatially separate the transfected from untransfected cells. Trypsinized and collagenase-treated cells were allowed to adhere to micropatterns for 1 hour. Cells were then transfected with the three mutant Rabs and received AA for 8 hours during the 9-hour course of transfection. Cells were then fixed and immunostained for extracellular collagen as well as GFP and total collagen to ensure that sufficient intracellular procollagen stores were being generated. Extracellular collagen was clearly visible in untransfected cells, however interestingly it appeared as punctate aggregates (Figure 9A–C), compared to the diffuse meshwork observed in spread cells (Figure 3). Expression of DN Rab1, 3d or 27b consistently resulted in reduced extracellular collagen levels, despite apparent normal intracellular procollagen production (Figure 9A–C). Image J quantification of extracellular protein collagen levels from merged Z-planes showed a significant reduction in extracellular collagen in cells expressing all three mutant Rabs, compared to untransfected control cells (Figure 9D–F).

## Discussion

OBs are unique collagen producers that show both increased collagen production and cell differentiation in response to agents like AA. AA has been shown to increase collagen mRNA production (1.3–1.8 fold), secretion rate of collagen, and enhance collagen matrix formation [46]. Thus OBs serve a unique model to study basal collagen secretion as well as large-scale collagen export. In resting OBs that undergo basal collagen secretion, we observed a dense accumulation of collagen that largely resides in the ER. Upon AA exposure, the collagen rapidly moves into the Golgi and appears on the cell surface in the course of a few hours.

We observed a rapid and specific up-regulation of known biosynthetic Rabs within hours of AA stimulation. Total Rab protein levels are likely elevated because modulation of the GTPase activity of the basal, pre-existing Rabs is presumably not sufficient to accommodate the massive transport of procollagen in AA-stimulated OBs. Rab GTPase mRNA expression is known to be regulated by several different kinases, including the ERK and p38 MAPK pathways [47] and AA has been shown to induce activation of protein kinase p38 MAPK [48] and promote expression of other genes such as OCN [49], Osx [50], procollagen [51]. The coordinated up-regulation of both cargo and trafficking machinery is likely essential for massive collagen deposition by AA-stimulated OBs.

We observed a near abolishment of procollagen protein levels in cells that were transfected with the DN Rab constructs overnight. While OBs may be particularly sensitive to alterations in intracellular trafficking, this data suggests that caution should be exercised when manipulating Rabs within the biosynthetic pathway. Mutant expression of Rab3d in differentiating OBs resulted in a reduction in AHA levels (indicating the total protein synthesis) following overnight transfection time periods. Although the AHA assay does not indicate the dynamic turnover of proteins, this reduction in protein synthesis due to mutant Rab3d expression is an interesting observation that has not been described in other cell types. Interestingly, treatment of fibroblasts from mouse models of Osteogenesis Imperfecta with BFA to block ER to Golgi transport of procollagen resulted in a reduction in procollagen production and proteasome-related degradation of procollagen in the cytosol [11], [52]. The major pathway for intracellular protein degradation is the ubiquitin-proteasome pathway [53] and both ERAD and Golgi-associated degradation (GAD) pathways are associated with proteasomes [54]. Treatment of Rab3d-DN-GFP expressing cells with MG132 rescued the intracellular and extracellular collagen level intensities. This raises the possibility that sustaining an active state of translation (e.g. procollagen biosynthesis) requires constant proteasomal activity. Our findings vary from previous studies where experimentally suppressing protein degradation in the cell caused reduced ribosome biogenesis and protein synthesis [55]. A large number of proteasomes compete with ribosomes to physically associate with the ER membrane [56]. For the purpose of this work, how specific mutant Rab GTPases accelerate the turnover of intracellular protein (procollagen) synthesis, at what point they regulate protein expression, and how they act on the proteasome pathway remains unknown.

**Figure 10:**
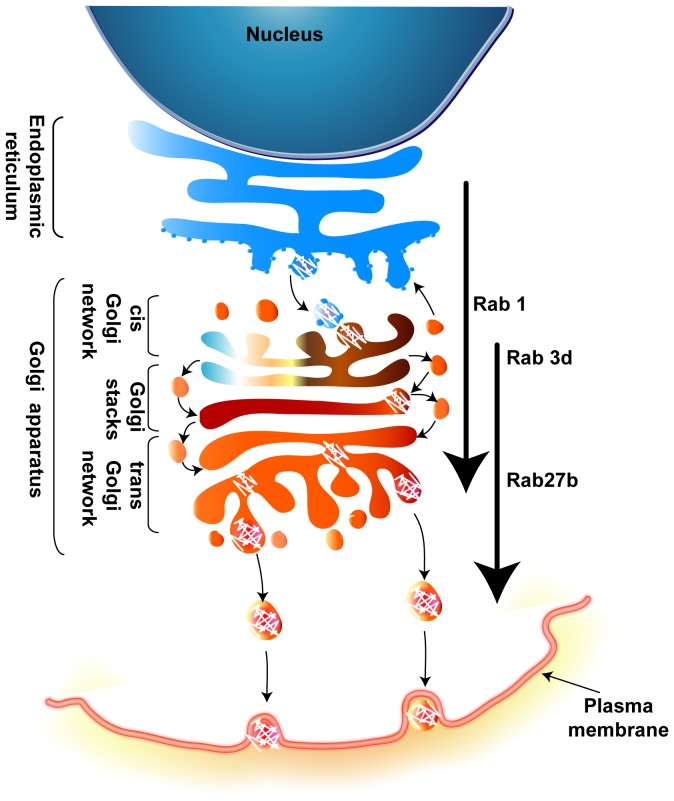
Proposed model. Following AA stimulation, OBs will begin to differentiate, synthesize and secrete collagen type I amongst other bone matrix proteins. The movement of procollagen from the ER to Golgi to PM is Rab GTPase dependent, namely via Rab1, Rab3d, and Rab27b.

Overnight transfection with mutant Rab constructs removed all intracellular collagen and was not useful for analyzing procollagen trafficking. However, moderate short-term expression of mutant Rabs did not activate negative feedback loops to the same extent, although some cells did show reduced total procollagen which may reflect differences in construct expression in transiently transfected cells. However, many transfected cells had detectable procollagen production and from these cells we were able to extract some information about procollagen translocation machinery in these cells. Rab1 is known to bind to effectors and facilitate vesicle budding from the ER and transport to Golgi [57] and the procollagen trafficking defects we observed in OBs are in agreement with Rab1 studies in several other cell types [58], [59]. In this work, we also identified Rab3d and Rab27b as Rab isoforms that were specifically up-regulated in differentiating OBs. AA-stimulation of OBs did not cause any change in mRNA levels of Rab27a, which has 71% identity with Rab27b at the amino acid level, and can compensate for Rab27b in some instances [60], [61]. In other cell types, Rab3d and 27b have been almost exclusively involved in post-Golgi trafficking of vesicles to the PM [39], [62], [63]. Expression of wild-type GFP chimeras showed strong cis-Golgi targeting for all three of the Rabs we examined, which was particularly pronounced in AA-stimulated OBs. While this is expected for Rab1, it is interesting that Rab3d and 27b also co-localize with cis-Golgi markers. Immunofluorescent analysis of Rab3d-GFP in Goblet cells also showed a recruitment of this Rab to the cis-Golgi [29] indicating that the characteristic ‘exocytic’ Rabs may function more upstream in highly secretory cells. Unlike other ubiquitous Rab GTPases, Rab3 and Rab27 are close homologous isoforms [64]. Dominant negative Rab constructs exert their inhibitory effects via sequestration of GEFs [65], [66], [67], [68] and Rab3d, Rab27b and Rab27a share a common GEF, Rab3GEP [69]. Thus the use of these dominant negative constructs may simultaneously impact multiple Rab GTPases. However, we have several lines of evidence that Rab3d and 27b do not play redundant roles in OBs. We observed dispersed procollagen containing vesicles in OBs expressing mutant Rab27b that did not co-label with ER or Golgi markers that could potentially represent failed secretory vesicles. This is interesting as it suggests the presence of a post-Golgi compartment of procollagen, indicating that this protein does not move completely through a cisternal maturation mechanism in OBs. Expression of Rab3d-DN-GFP did not produce a similar build-up of procollagen containing vesicles in the cytoplasm, suggesting that Rab27b acts more downstream than Rab3d in osteoblasts (Figure 10). Interestingly, expression of mutant Rab3d in particular, had a more profound effect on procollagen levels in AA-treated OBs than control unstimulated cells. Therefore it appears that basal collagen trafficking in resting OBs uses different mechanisms than in AA-stimulated cells. This makes sense as differentiating OBs secrete a remarkably higher amount of collagen [46] compared to resting cells therefore specialized mechanisms must be assembled to accommodate this bolus of mobilized procollagen.

Similar to Rab1, we observed a marked reduction in extracellular collagen when DN Rab3d and 27b were expressed in AA-stimulated OBs. For these analyses we exploited CYTOO micropatterned slides to spatially separate and easily discern the precise secretory cells and their associating secreted collagen. An interesting extracellular collagen phenotype pattern observed in mutant cells plated on CYTOO slides was the disappearance of long collagen fibrils extending and connecting differentiating cells. Normally, confluent differentiating OBs on glass coverslips secrete characteristic extensive collagen fibrils and their absence in spatially separated cells may suggest a mechanistic link between cell-cell contact sites and proper collagen fibrillogenesis [70]. Our immunofluorescence analysis of external and total collagen in spread OBs also did not reveal the presence of fibripositors in this cell type. Thus different mechanisms are likely at play to move procollagen from the Golgi to the cell surface in the bone-producing OBs versus fibroblasts. The identification and characterization of intracellular molecules controlling procollagen processing and trafficking in OBs will be important for developing therapies for diseases driven by aberrant collagen processing and secretion.

## Materials and Methods

### Reagents and Antibodies

Fetal bovine serum (FBS) and RPMI 1640 containing 25 mm HEPES (HPMI) were purchased from Wisent Inc. (St. Bruno, QC, Canada). Alpha minimal essential medium (α-MEM) without AA was from Gibco (Carlsbad, CA, USA). 0.05% Trypsin/0.53 mM EDTA was purchased from Multicell. Antibodies were obtained as follows: Monoclonal mouse anti-GM130 from BD Transduction Laboratories™ (San Jose, CA, USA), mouse anti-PDI from Assay Designs, rabbit polyclonal anti-MMP-9 from Enzo® Life Sciences (Plymouth, PA), polyclonal rabbit anti-GFP antibody from Abcam (Cambridge, UK), and rabbit polyclonal anti-type I collagen from Cedarlane (ON, Canada). Collagenase Type I was from Bioshop (Burlington, Canada). All fluorescently labelled secondary antibodies and horseradish peroxidase-conjugated secondary antibodies were purchased from Jackson ImmunoResearch Laboratories (West Grove, PA, USA). The Pierce chemiluminescence kit was obtained from Thermo Scientific (Rockford, IL, USA). BFA, 4′-6-Diamidino-2-phenylindole (DAPI), phalloidin, and Click-iT metabolic labelling reagents for proteins were from Invitrogen (Carlsbad, CA, USA). All other reagents were purchased from Sigma-Aldrich (St Louis, Missouri, USA).

### RNA Extraction and RNA Expression Profiling by Affymetrix Microarray

To determine which Rab GTPases are endogenously up-regulated during MC3T3-E1 OB differentiation, we utilized Affymetrix microarray strategies. Total RNA from control and 5-day AA-treated (50 µg/ml) MC3T3-E1 OB cells grown in 6-well plates in triplicate was isolated with Trizol Reagent (Invitrogen, Carlsbad, USA). After this, the aqueous phase (containing the RNA) was combined with 70% ethanol, loaded onto an RNeasy column (Qiagen, Valancia, USA) and purified according to manufacturer's instructions. RNA integrity was verified on an Agilent® 2100 Bioanalyzer (Agilent Technologies, Santa Clara, CA, USA). The hybridized MOE430.20 microarray gene chip arrays were washed and stained according to the manufacturer's instructions and scanned using an Affymetrix Scanner 3000 at the Center for Applied Genomics at SickKids (Toronto, ON, Canada). The resulting .CEL files were analyzed numerically using Partek Genomics Suite Software and normalized using quantile normalization to compensate for systematic technical differences between chips and the signals from multiple probes. Data were then corrected for background non-specific probe affinities to the gene chip using GC Robust Multi-array Average (GC-RMA) to obtain a single gene expression value. Principal component analysis (PCA) was used to survey gene variation across undifferentiated control and 5-day AA differentiated triplicates and variations across the replicates separately. The numeric values presented in Figure 1 are the mean of the triplicate experiments ± standard deviation for a partial list of up-regulated Rab GTPases.

### Microarray Data Validation by Quantitative RT-PCR

Total mRNA from control, 6 hrs, and 5-day AA-treated cells grown in 6-well plates was extracted using the RNeasy kit (Qiagen, Valencia, CA) The integrity of total RNA was assessed by a NanoDrop®ND-100 Spectrophotometer (Thermo Fisher Scientific, Inc., Waltham, USA) and by a denaturing agarose gel. cDNA was synthesized from 1 µg total RNA, using the SuperScript III First-Strand Synthesis SuperMix for qRT-PCR from Invitrogen (Invitrogen,Carlsbad, USA) according to manufacturer's protocol. A serial dilution curve experiment was performed for every gene of interests to establish a standard curve. The specific primers for each gene were synthesized according to standard procedures in the literature:

Rab1-F, 5′- TGACATGTCCAGCATGAATCCCGA -3′

Rab1-R, 5′- CCTGGCCTGCTGTGTCCCAT -3′

Rab3d-F, 5′- GATTTGGGACACAGCAGGCCAGG -3′

Rab3d-R, 5′- CAAGATCATCGGCGAGCCTCC -3′

Rab27b-F, 5′- TTCCAGAGGAGAAGCGGACGCA -3′

Rab27b-R, 5′- GGCACCTCCAAACGCTTCCAGAT -3′

Rab27a-F, 5′- TCGGATGGAGATTACGATTACCT -3′

Rab27a-R, 5′- TTTTCCCTGAAATCAATGCCCA -3′

Rab7-F, 5′- AAGCCACAATAGGAGCGGAC -3′

Rab7-R, 5′- AGACTGGAACCGTTCTTGACC -3′

COL1A1-F, 5′- CATGTTCAGCTTTGTCCAGGT -3′

COL1A1-R, 5′- GCAGCTGACTTCAGGGATGT -3′

GAPDH-F, 5′- TCAACGACCCCTTCATTGAC -3′

GAPDH-R, 5′- ATGCAGGGATGATGTTCTGG -3′

Identical volumes of cDNA were loaded for all samples, and samples were run in triplicate. PCR conditions were run as follows: initial incubation step at 95°C for 3 minutes, 40 cycles consisting of 30 seconds at 95°C, 30 seconds at 55°C, and 30 seconds at 72°C using a DNA Engine Opticon System (MJ Research Incorporated), and 25 µl total reaction volumes containing cDNAs, SYBR Green I dye (BioRad) and primer sets. Real time reverse transcription-PCR was carried out using the MyIQ iCycler system (Bio-Rad) according to the manufacturer's instructions. After the last cycle, a dissociation curve was generated by first collecting fluorescent signals at 55°C and taking measurements at 2 second intervals with increasing temperature until it reached 95°C. We confirmed a linear range of amplification for all primers and products and melting curves were used to verify the absence of nonspecific PCR products.

The data was calculated using the comparative Ct (Cycle threshold) method (2-[delta][delta]Ct method) by which [delta][delta]Ct = [delta]Ct sample- [delta]Ct reference [71], [72], [73]. Three independent experiments were performed for each sample and in triplicate, and the corresponding results were normalized to housekeeping genes and expressed as mean ± S.E.

### Cell Culture Maintenance

The mouse calvarial pre-osteoblastic (MC3T3-E1) cell line subclone 4 was obtained from the American Type Culture Collection (Manassas, VA) and maintained at 37°C supplied with 5% CO_2_ in α-MEM (without AA) supplemented with 10% heat-inactivated FBS with or without 50 µg/ml AA and renewed every 2 days. For all experiments, undifferentiated cells grown to 60–80% confluence were passaged using 0.05% trypsin and 1 µg/ml collagenase to remove pre-existing extracellular collagen and then plated in 6-well plates (Starstedt Inc, Montreal, Canada) with 25 mm glass coverslips for immunofluorescence studies. For extracellular collagen analysis, cells were plated on poly-L-lysine adhesive disc-shaped micro-patterned coverslips (CYTOO Cell Architects, Grenoble, France). For western blotting and RNA extraction, cells were grown in 6-well plates without coverslips. MC3T3-E1 cells were transiently transfected using FuGENE HD (Roche Diagnostics, Laval, Quebec) or TransIT2020 (Mirus Bio LLC, Madison, USA) according to the manufacturer's instructions. Cells were differentiated with 50 µg/ml of AA either overnight or for shorter periods of time.

### Immunofluorescent Staining and Microscopy

Cells were fixed with 4% paraformaldehyde (PFA) (Canemco Inc., Lakefield, Quebec, Canada) and permeabilized with 0.1% Triton X-100 with 100 mM glycine in PBS for 20 minutes at RT. They were then blocked in 5% FBS in PBS for 1 hour at room temperature (RT) prior to staining with primary antibodies. The primary antibody dilutions used were: sheep polyclonal anti-type I collagen (1∶200); mouse monoclonal GM130 and PDI (1∶200); Rabbit polyclonal anti-MMP-9 (1∶1000) and mouse monoclonal anti HSP70 antibody (1∶200) for 1 hour. Cells were subsequently washed with PBS and incubated with compatible fluorescent-conjugated secondary antibodies (1∶1000) and/or phalloidin (1∶500) for detection. The cells were then washed in PBS and incubated with DAPI (1∶10,000) for 10 minutes for nuclear staining prior to mounting. Images were taken using either an inverted Axio-Observer Z1 microscope equipped with DIC and epifluorescence optics or a Zeiss LSM 510 confocal microscope under the 40× oil immersion objective (Carl Zeiss Microimaging Inc., Germany). Images were post-processed using Axiovision and Adobe Photoshop CS (Adobe Systems, Inc.). For external collagen immunostaining, all the steps are similar to those explained above except that immunostaining was performed without permeabilization. After fixation, cells were incubated with blocking buffer and subsequently with primary collagen and secondary antibodies for 1 hour. To label both extracellular secreted collagen, as well as intracellular procollagen, cells were then permeabilized and stained with our collagen antibody as described. Confocal Z-stacks for colocalization studies were acquired on a Zeiss LSM 510 confocal microscope.

### Western Blot Analysis

To determine the levels of collagen type I in control, 1 and 5 days AA-treated MC3T3-E1 cells, and BFA treated cells, western immunoblotting was performed on the cell lysates and their respective conditioned media. Lysates were prepared by solubilizing the cells in RIPA buffer (10 mM Tris-HCl, 150 mM NaCl, 1% Triton X-100, 0.1% SDS, 1 mM EDTA, pH 7.4) containing 2 µg/ml phosphatases and protease inhibitors. The protein concentration was determined by using a Bio-Rad protein assay kit and Biotek Synergy HT plate reader. 20 and 70 µg of total protein extracted from cell lysates and conditioned media was boiled in sample buffer consisting of 2% SDS, 10% glycerol, 60 mM Tris, pH 8.8, and 0.001% bromophenol blue. Protein from cell lysates and conditioned media were electrophoresed in 8% SDS-polyacrylamide gels and transferred to nitrocellulose membrane (Millipore, Bedford, MA) according to the manufacturer's directions. The membranes were blocked in T-TBS (0.1% Tween 20 in 20 mM Tris-HCl, pH 7.6, 137 mM NaCl) with 5% skim milk powder overnight. After washing in T-TBS, the blots were incubated with anti-collagen antibody (1∶1000) for 1 hour and anti-rabbit horseradish peroxidase-conjugated secondary antibody (1∶1000) for 1 hour. Bands were detected using the Pierce chemiluminescence kit.

### Plasmid Construction

Total RNA was isolated using the RNAeasy kit (Qiagen, Valancia) according to manufacturer's instructions and reverse transcribed into cDNA using one-step SuperScript III kit (Invitrogen). To generate GFP–Rab3d fusion protein, mouse Rab3d cDNAs were obtained by RT-PCR on total mouse RNA isolates using the following primers:

Rab3d-F, 5′- ATTCTCGAGATGGCATCCGCTAGT -3′

Rab3d-R, 5′- ATTGAATTCCTAACAGCTGCAGCTGCT -3′

cDNAs encoding the mouse Rab3d sequence were subcloned into pEGFP-C1 expression vector (Clontech). Site-directed mutagenesis of GFP-Rab3d was used to produce S22N point mutation in Rab3d and was carried out according to instructions in the QuickChange site-directed mutagenesis kit (Stratagene). Mutant cells have shown to exhibit impaired GTP binding and show a cytosolic/nuclear distribution [74]. Plasmid DNA constructs were transformed into MAX Efficiency® DH5α™ Competent Cells (Invitrogen Canada Inc.) for propagation, and purified by EndoFree® Plasmid Maxi kit (QIAGEN Inc., Mississauga, Ontario), according to manufacturer's instructions. Sequences of all constructs were verified by automated sequencing (Sickkids, Toronto, Ontario).

### Nascent Protein Synthesis Labelling

Nascent collagen protein synthesis was detected with Click-iT AHA (L-azidohomoalanine) protein synthesis labeling system (Invitrogen), which has been shown to be effective in labeling newly synthesized proteins in mammalian neurons [44]. Cells were passaged on 25-mm glass coverslips using trypsin and collagenase and transfected with Rab3d-DN for 6 and 24 hours and differentiated with AA for 6 hours (±6 hour or 24 hour MG132). Two hours prior to fixation, they were incubated for 1 hour in 1 ml of serum/methionine free AMEM media and incubated with methionine-free media supplemented with 50 µM AHA for another hour. Cells were then fixed with 4% PFA, washed with 3% BSA, permeabilized with 0.25% TritonX-100 in PBS for 20 minutes, and AHA incorporation was visualized using 50 µM Alexa Fluor 555 alkyne triethylammonium (A20013, Invitrogen) diluted in the manufacturer recommended reaction buffer (C10269, Invitrogen) and incubated for 30 minutes as described in manufacturer's protocol. Cells were then mounted for visualization with Axio-Observer Z1 fluorescence microscope.

### Statistical Analysis

The PCA plot was used to identify the predominant gene expression pattern trends in the data and summarize and reduce the correlations between data from undifferentiated control and 5day AA-treated conditions into new axes or principal components (PCs) with percents of variation. The triplicate data circles from undifferentiated control samples are positively correlated in the oval net and independently separate from differentiated dataset on the right oval net. The closer to the circles, the higher is the coefficient of correlation between variables. The PCA plot represents the normalized, standardized, and averaged results of the triplicate experiments ± standard deviation.

Paired t-tests were used to evaluate differences between treatments. A critical p-value of 0.01 was considered as the criterion to select a significant fold change in gene expression between treatments. The amount of mRNA for qRT-PCR studies was quantified by determining the point at which the fluorescence accumulation entered the exponential phase (C_t_), and the C_t_ ratio of the target gene to GAPDH was calculated for each sample. Data were analyzed using Excel (Microsoft) and correspond to means ± SE. Data were considered significant if p<0.05. Results for PCR analysis were expressed as the mean ± S.E. and analyzed statistically by one-way analysis of variance and a post hoc Bonferroni test.

To measure the number of Rab-positive procollagen containing vesicles, we measured the sum of intensities of colocalizing pixels relative to the overall sum of pixel intensities above the threshold (or background), also referred to as weighted colocalization. These were determined confocal images of untreated and AA-treated MC3T3-E1 cells. The advantage of a weighted colocalization coefficient is that differences in pixel intensity are taken into account (i.e. not all pixels contribute equally to the final colocalization coefficient value). Procollagen containing vesicles showing a weighted coefficient greater than or equal to 0.4 with Rab-positive vesicles were scored as positive within each cell/treatment. The sums of colocalizing pixels were averaged in thirty cells and p<0.05 was considered significant.

To determine the effect of overnight expression of DN Rabs on procollagen levels, fluorescent images of 100 transfected cells (Rab1-DN-GFP, Rab3d-DN-GFP, Rab27b-DN-GFP) were compared to 100 untransfected cells in the same field of view, respectively. To reduce operator bias, the control non-differentiated and differentiated cells were blinded. The images from transfected cells were visually inspected and scored with reduced collagen levels depending on their staining intensity compared to their adjacent untransfected cells in the same field of view taken with the same light intensity. Thus, the Y-axes in Figure 8 represents the % of Rab DN transfected cells showing differential collagen levels compared to untransfected control cells.

To determine the effects of acute expression of dominant negative constructs of Rab1, 3d, and 27b, control and 6 hour AA-treated cells were transfected with the respective mutant constructs for 6 hours, fixed, and immunostained with collagen, ER, and Golgi markers. The 6 hour GFP signal was amplified using immunostaining with an anti-GFP antibody. The intracellular collagen levels were inspected in 100 cells and scored into four categories of normal, low, high, and no collagen. The Y-axis represents the percentage of Rab mutant cells displaying a particular collagen phenotype, compared to untransfected control cells within the same field of view. The graphs only show data from cells expressing high levels of mutant Rab-GFP constructs; the low and mid-expressing cells were not included in these analyses to ensure that a dominant negative effect from overexpression was being observed. To determine the collagen localization within the biosynthetic pathway, the collagen levels in ER and Golgi immunostained cells in 100 cells expressing high levels of mutant Rabs for 6 hours were assessed and compared to untransfected control cells. Cells were scored in two categories of increased or decreased collagen levels in each organelle based on their intensities with respect to untransfected cells in the same field of view from epifluorescent images. Quantified data correspond to means ± SE in 3 independent experiments. Significance was represented as p<0.05.

The extracellular collagen intensity levels were measured in cells that were transfected with DN Rab constructs for 9 hours and differentiated with AA for 8 hours. Image J was used to individually draw a circle around the CYTOO chip micropatterns and measure the intensities of extracellular collagen staining in 50 cells. The final immnofluorescent images for extracellular collagen measurements were obtained with from Z-stacks compressed into one image using extended focus on Zeiss Axiovision software. Data are presented as the mean ± SEM. All experimental data were repeated in triplicates. Difference in p-values<0.05 were considered statistically significant.

## Supporting Information

Figure S1 **qRT-PCR validation of mRNA expression levels of Rab7 and Rab27a in 5-day AA-treated cells compared to undifferentiated control cells.** (A) Rab7 mRNA expression with the Y-axis representing the fold change in AA-stimulated OBs compared to controls in triplicate experiments. (B) Rab27a mRNA expression levels in 5-day AA-stimulated OBs compared to control cells. Neither gene expression was significantly different in AA-treated cells, compared to control cells, * p>0.05.
(DOC)
