# Supplementary material for: Rab GTPase Mediated Procollagen Trafficking in Ascorbic Acid Stimulated Osteoblasts
Source: PLoS One. 2012 Sep 26;7(9):e46265. doi: 10.1371/journal.pone.0046265 (PMC3458846; doi:10.1371/journal.pone.0046265)
Supplement: Figure S1 — qRT-PCR validation of mRNA expression levels of Rab7 and Rab27a in 5-day AA-treated cells compared to undifferentiated control cells. (A) Rab7 mRNA expression with the Y-axis representing the fold change in AA-stimulated OBs compared to controls in triplicate experiments. (B) Rab27a mRNA expression levels in 5-day AA-stimulated OBs compared to control cells. Neither gene expression was significantly different in AA-treated cells, compared to control cells, * p>0.05. (DOC) [file pone.0046265.s001.doc]

**Supporting information legend**

Figure S1. qRT-PCR validation of mRNA expression levels of Rab7 and Rab27a in 5-day AA-treated cells compared to undifferentiated control cells. (A) Rab7 mRNA expression with the Y-axis representing the fold change in AA-stimulated OBs compared to controls in triplicate experiments. (B) Rab27a mRNA expression levels in 5-day AA-stimulated OBs compared to control cells. Neither gene expression was significantly different in AA-treated cells, compared to control cells, * p > 0.05.
